# Supplementary material for: Deep ocean warming-induced El Niño changes
Source: Nat Commun. 2024 Jul 23;15:6225. doi: 10.1038/s41467-024-50663-9 (PMC11266720; doi:10.1038/s41467-024-50663-9)
Supplement: Supplementary file 1 — Supplementary Information [file 41467_2024_50663_MOESM1_ESM.pdf]

# Supplementary Information for

## Deep ocean warming-induced El Niño changes

Geon-Il Kim[1], Ji-Hoon Oh[1], Na-Yeon Shin[1], Soon-Il An[2], Sang-Wook Yeh[3], Jongsoo Shin[4] and Jong-Seong Kug[1,5,*]

[1]School of Earth and Environmental Sciences, Seoul National University, Seoul, South Korea

[2]Department of Atmospheric Sciences, Yonsei University, Seoul, South Korea

[3]Department of Marine Sciences and Convergent Technology, Hanyang University, Ansan, South Korea.

[4]Woods Hole Oceanographic Institution, Woods Hole, Massachusetts, United States of America

[5]Interdisciplinary Program in Artificial Intelligence, Seoul National University, Seoul, South Korea

*Corresponding author: Jong-Seong Kug (email: jskug1@gmail.com)

## Supplementary Methods

### Bjerknes stability (BJ) index

The BJ index was constructed by estimating the sensitivity coefficients in the approximated form of the linearized equation for ocean-temperature tendency (Eq. 1), in accordance with previous studies [1–3].

$$BJ = -\underbrace{\left(a_1\frac{\langle\Delta\bar{u}\rangle_E}{L_x} + a_2\frac{\langle\Delta\bar{v}\rangle_E}{L_y}\right)}_{MA}\underbrace{-\alpha_s}_{TD}\underbrace{+\mu_a\beta_u\langle-\frac{\partial\bar{T}}{\partial x}\rangle_E}_{ZA}\underbrace{+\mu_a\beta_w\langle-\frac{\partial\bar{T}}{\partial z}\rangle_E}_{EK}\underbrace{+\mu_a^*\beta_h\langle\frac{\bar{w}}{H_1}\rangle_E\, a_h}_{TH} \quad (1)$$

where $(T, u, v, w)$ represent the ocean temperature and three-dimensional current anomalies, respectively. The variable with the over-bar represents the climatological mean state for the analysis period. The ocean temperature and horizontal currents are averaged from the surface to a depth of 50m, and the subsurface temperature and vertical currents are values at 55 m and 50 m, respectively. Where $\langle \square \rangle_E$ and $[\square]$ denote the average area of the eastern Pacific (150˚W–90˚W, 5˚S–5˚N) and the equatorial Pacific (120˚E–90˚W, 5˚S–5˚N), respectively. $L_x$ and $L_y$ are the longitudinal and latitudinal length of the equatorial box, respectively. The coefficients $\beta_u$, $\beta_w$, and $\beta_h$ indicate the sensitivity of the response of oceanic variables (e.g., $\langle u\rangle_E$, $\langle h\rangle_E - [h]$, and $\langle H(\bar{w})w\rangle_E$) in relation to wind stress forcing over the eastern Pacific. Here, $H(x)$ is a step function that considers only upward vertical motion, and $h$ represents the thermocline depth which is substituted for sea surface height. The regression coefficients $\mu_a$ and $\mu_a^*$ represent the average wind stress over the eastern and equatorial Pacific, respectively, in relation to the eastern Pacific temperature. $\alpha_s$ represents the regression coefficient of the net heat flux of the eastern Pacific temperature. The coefficient $a_h$ measures the response of the subsurface temperature to thermocline depth, considering only the upward vertical motion. The definition of parameters and the associated response sensitivity coefficients are specified in Supplementary Table 1. The first two terms represent damping, which makes the BJ index negative, and are the effect of mean current (MA) and thermal (TD), respectively. The last three terms represent the zonal advection feedback (ZA), Ekman pumping feedback (EK), and thermocline feedback (TH), respectively.

**Supplementary Note**

**Robustness from CMIP6 models**

To strengthen the robustness of our findings, we further analyzed the 8 model simulations from the Carbon Dioxide Removal Model Intercomparison Project (CDRMIP)[4] of CMIP6, which has a similar experimental protocol to the simulation in this study. All of the model data are bilinearly interpolated onto a common 1°× 1° grid before analysis. The length of the recovery period (constant $CO_2$ forcing period) varies among models (Supplementary Table 2). Note that several models provide too short restoring period to quantify significant El Niño changes.

Supp. Figure 6a shows the difference in the temperature from the picontrol to the restoring period. The SST pattern in the MME is quite similar to the result of this study, implying that the deep ocean warming induces the El Niño-like warming in the restoring period. To check the changes in ENSO characteristics, we calculate the changes in the STD of the Niño3 SSTA from the picontrol to the restoring period (Supplementary Fig. 6b). Five of the eight models show an increase in the STD of the Niño3 SSTA, with four showing an increase of more than 8%, except for CNRM-ESM2-1, which is statistically significant. The MME values show a statistically significant increase of about 7%. Convective extreme El Niño increased in all models except the MIROC-ES2L model, although CanESM5 has no value as no convective El Niño occurred in either period (Supplementary Fig. 6c). In particular, the top four models with large increases in the Niño3 STD show a large increase in the frequency of convective El Niño events. The MME results indicate that the number of convective extreme El Niño events has been about double during the restoring period. The CDRMIP results are overall consistent with our main results, suggesting the robustness of our results, but a caution is needed for the short restoring period.

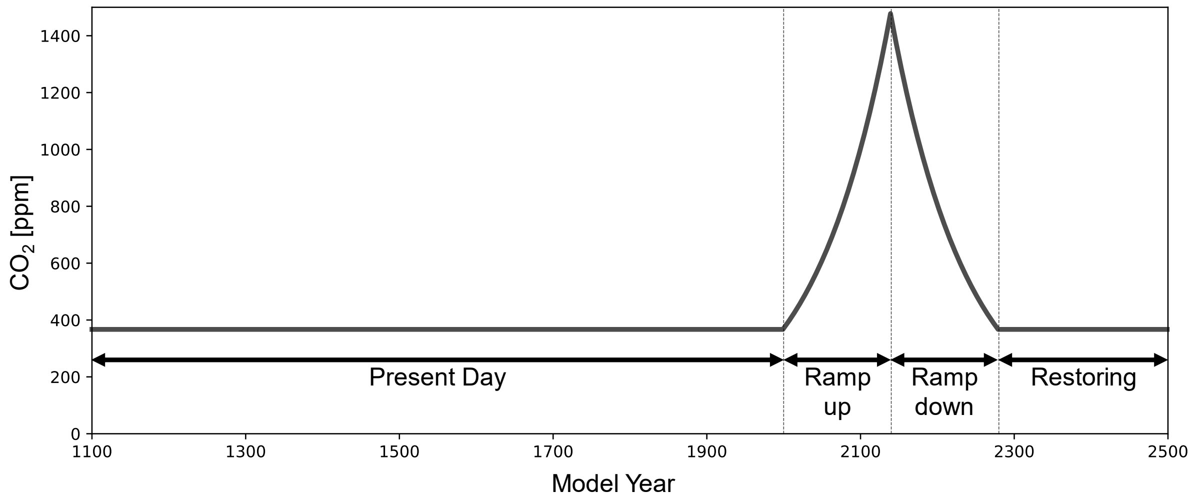


**Supplementary Fig. 1 | Idealized $CO_2$ scenario imposed in the Earth System Model experiment.** The black line indicates the $CO_2$ pathway with model year. Present day (PD, 1100-2000 years), ramp-up (2000-2140 years), ramp-down (2140-2280 years), and restoring period (2280-2500 years) were delineated on the figure.

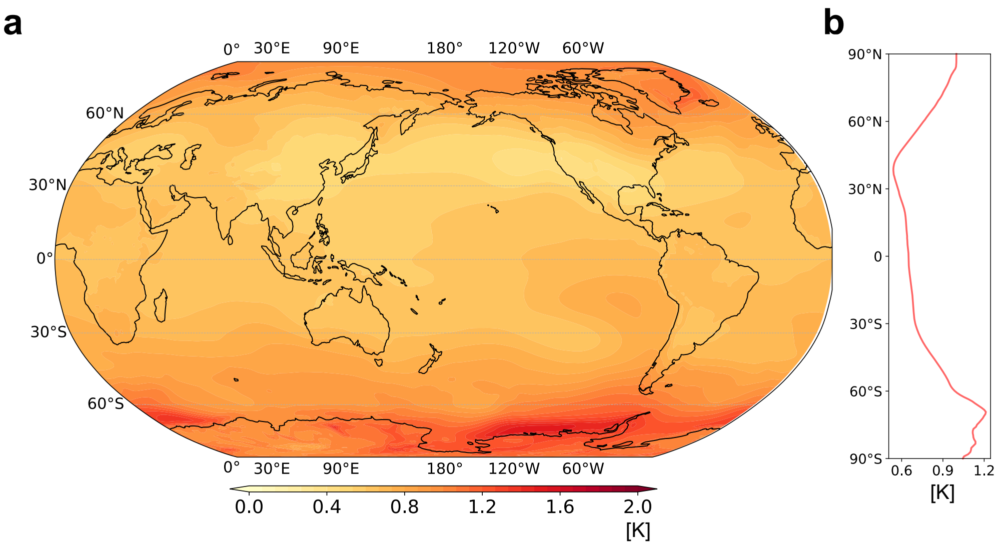


**Supplementary Fig. 2 | The changes in the air temperature.** The difference in **a** the spatial pattern and **b** the zonal averaged the air temperature from the PD period to restoring. The air temperature is vertically averaged from the surface to 300hPa.

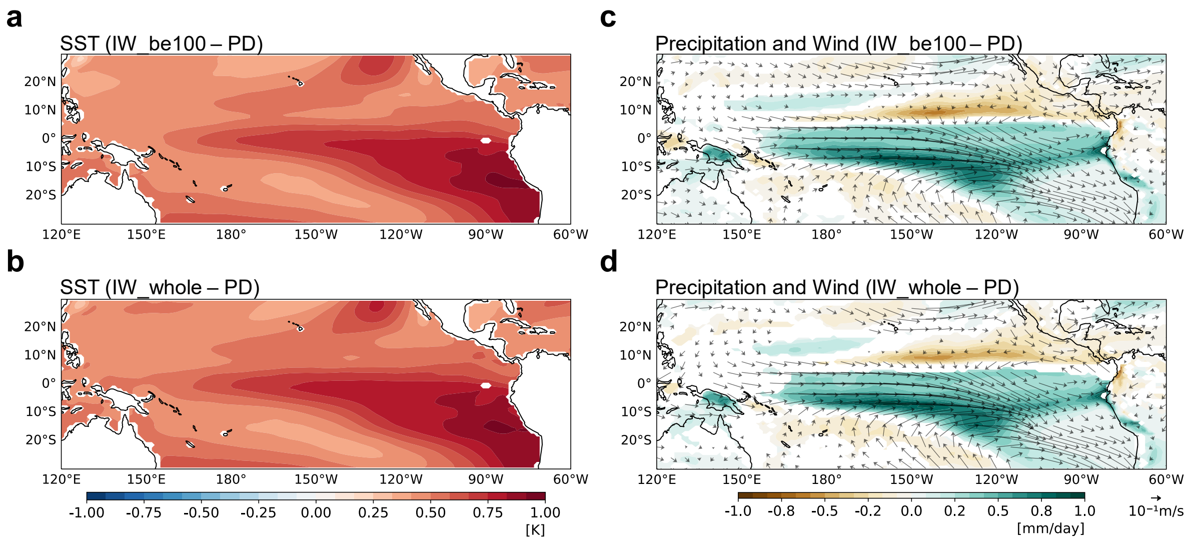


**Supplementary Fig. 3 | The changes in the tropical mean state.** The difference in **a** SST, **c** precipitation and wind at 850hPa between IW_be100 and the PD period. The difference in **b** SST, **d** precipitation and wind at 850hPa between IW_whole and the PD period. Only significant values at the 95% confidence level using the bootstrap test are shown.

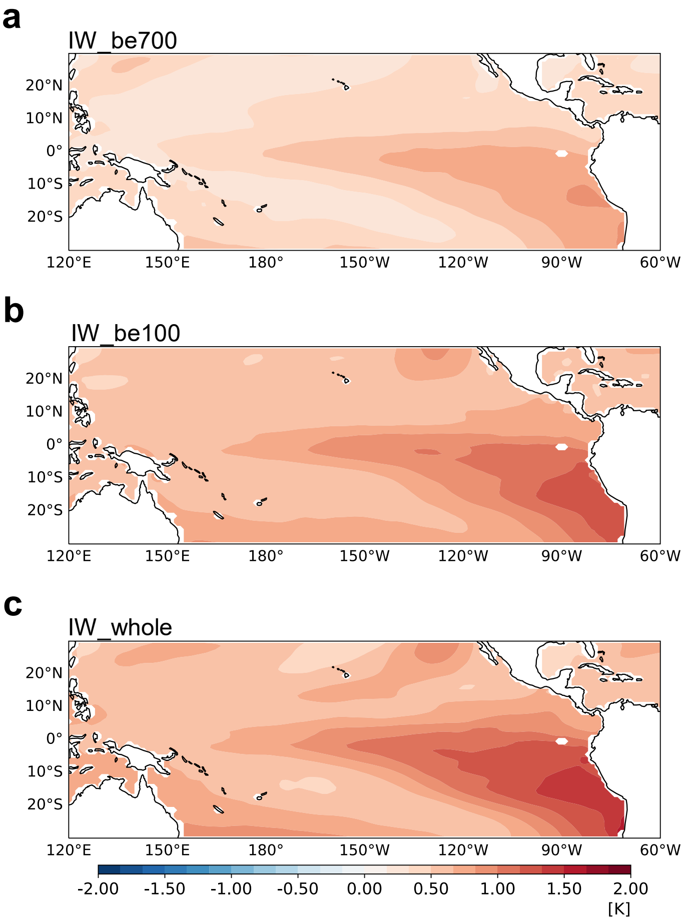


**Supplementary Fig. 4 | The initial warming pattern of IW experiment.** The difference in SST from the PD period to the initial five-year of **a** IW_be700, **b** IW_be100 and **c** IW_whole.

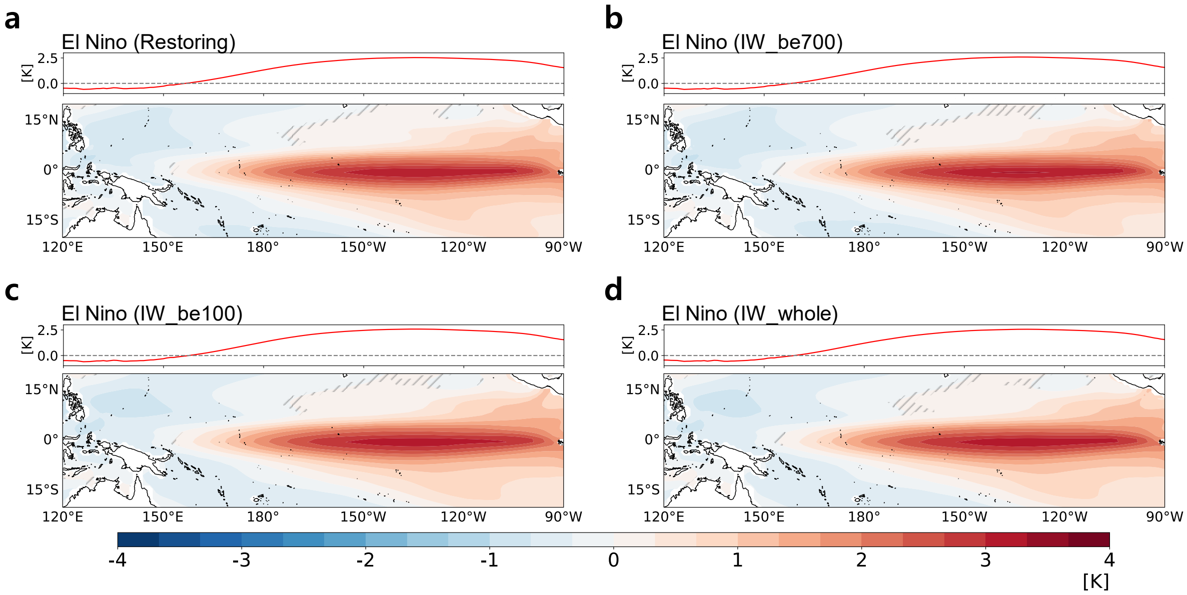


**Supplementary Fig. 5 | The spatial pattern El Niño.** The spatial pattern of El Niño (shading) and its equatorial average (5°S-5°N, red line) in **a** restoring, **b** IW_700 **c** IW_be100 and **d** IW_whole. Stippled areas are regions that are not significant at the 95% confidence level of Student's t-test.

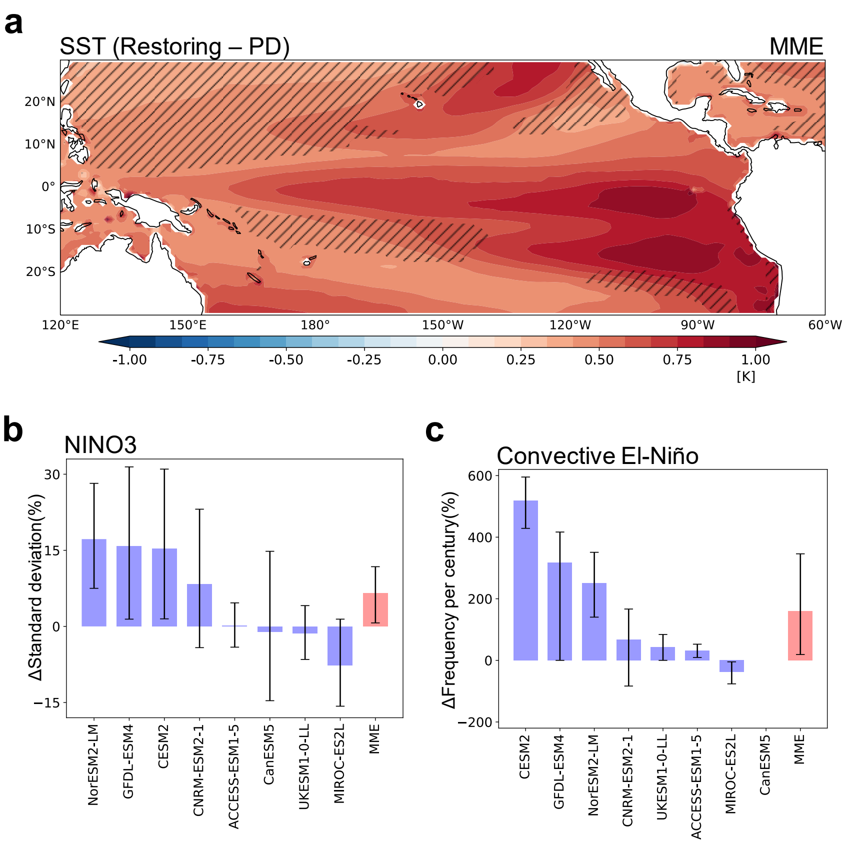


**Supplementary Fig. 6 | The changes in the tropical mean state and ENSO amplitude in CMIP6. a** The difference in SST between the restoring and PD period. **b** The difference in the STD of the DJF Niño3 SSTA and **c** the frequency of convective El Niño between the restoring and PD period. Error bars represent the 90% confidence interval using the bootstrap test. Stippled areas are regions that are not significant at the 90% confidence level for the differences of restoring and PD period using the bootstrap test.

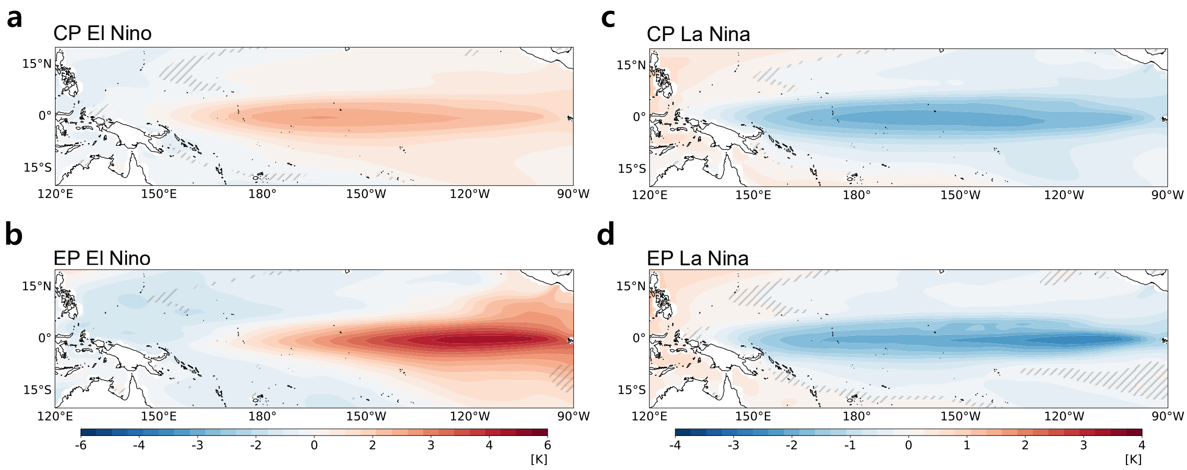


**Supplementary Fig. 7 | The spatial pattern of EP and CP ENSO.** The spatial pattern of **a** CP El Niño, **b** EP El Niño, **c** CP La Niña and **d** EP La Niña SSTA during the PD period. Stippled areas are regions that are not significant at the 95% confidence level of Student's t-test.

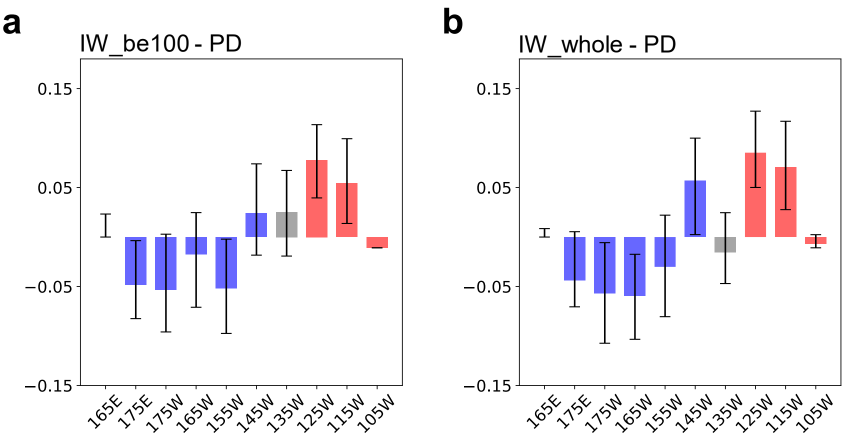


**Supplementary Fig. 8 | The changes in El Niño's flavor.** Histogram (normalized occurrences) of the difference in the centers of El Niño SSTA during DJF from the PD period to **a** IW_be100 and **b** IW_whole. Error bars represent the 95% confidence interval using the bootstrap test.

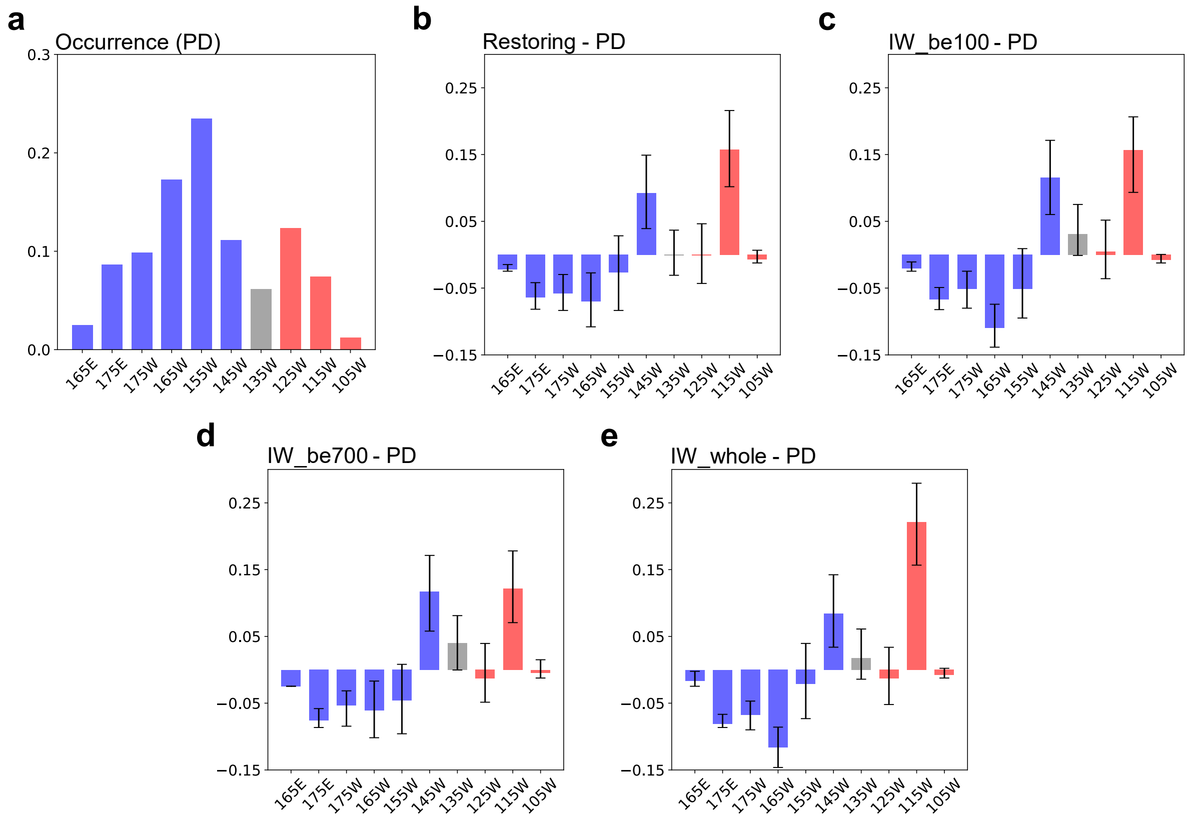


**Supplementary Fig. 9 | The changes in La Niña's flavor. a** Histogram (normalized occurrences) of La Niña SSTA centers during DJF for the PD period. As in **a** but for the difference from the PD period to **b** restoring, **c** IW_be100, **d** IW_be700 and **e** IW_whole. Error bars represent the 95% confidence interval using the bootstrap test.

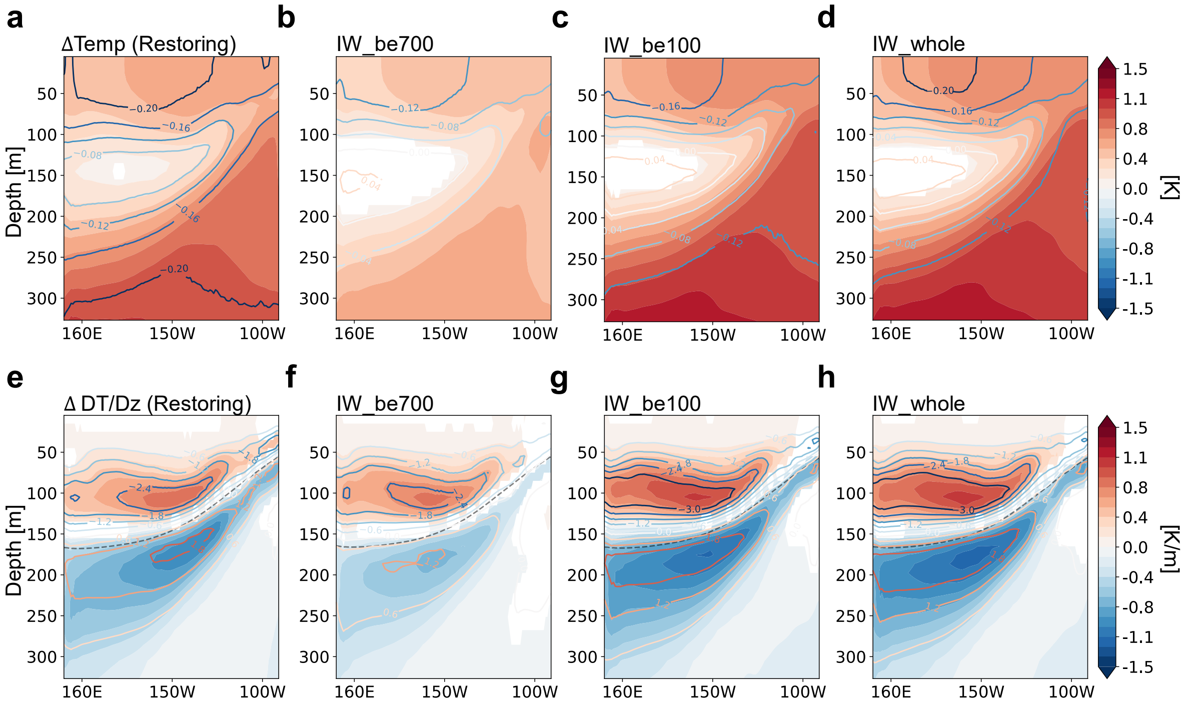


**Supplementary Fig. 10 | The changes in ocean temperature stratification.** The difference in the equatorial averaged (5°S–5°N) vertical climatological temperature and potential density from the PD period to **a** restoring, **b** IW_be700, **c** IW_be100 and **d** IW_whole. The difference in the equatorial averaged (5°S–5°N) vertical gradient of climatological temperature and potential density from the PD period to **e** restoring, **f** IW_be700, **g** IW_be100 and **h** IW_whole. The shading, contour and dashed line indicate the temperature, potential density and the depth of the thermocline, respectively, in each experiment. Temperature values that are significant at the 95% confidence level using the bootstrap test are shown.

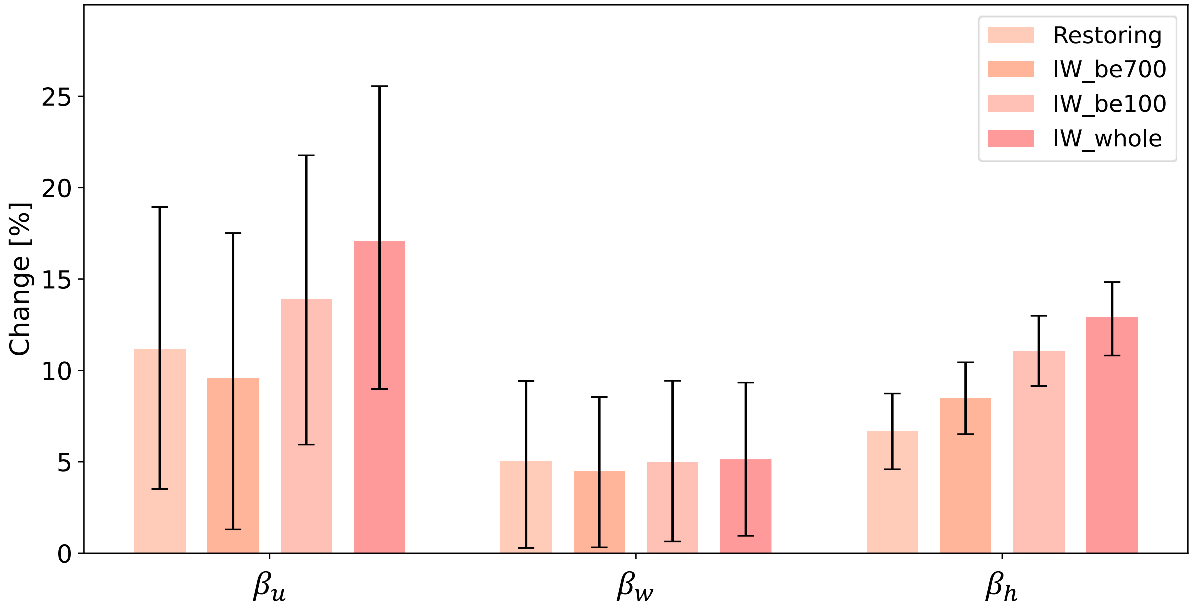


**Supplementary Fig. 11 | The changes in the current and thermocline response to wind stress.** The response of zonal currents, ocean upwelling and the zonal slope of the thermocline to wind stress forcing from the PD period to restoring, IW_be700, IW_be100 and IW_whole, respectively. Each value represents a percentage change from the PD period. Error bars represent the 95% confidence interval using the bootstrap test.

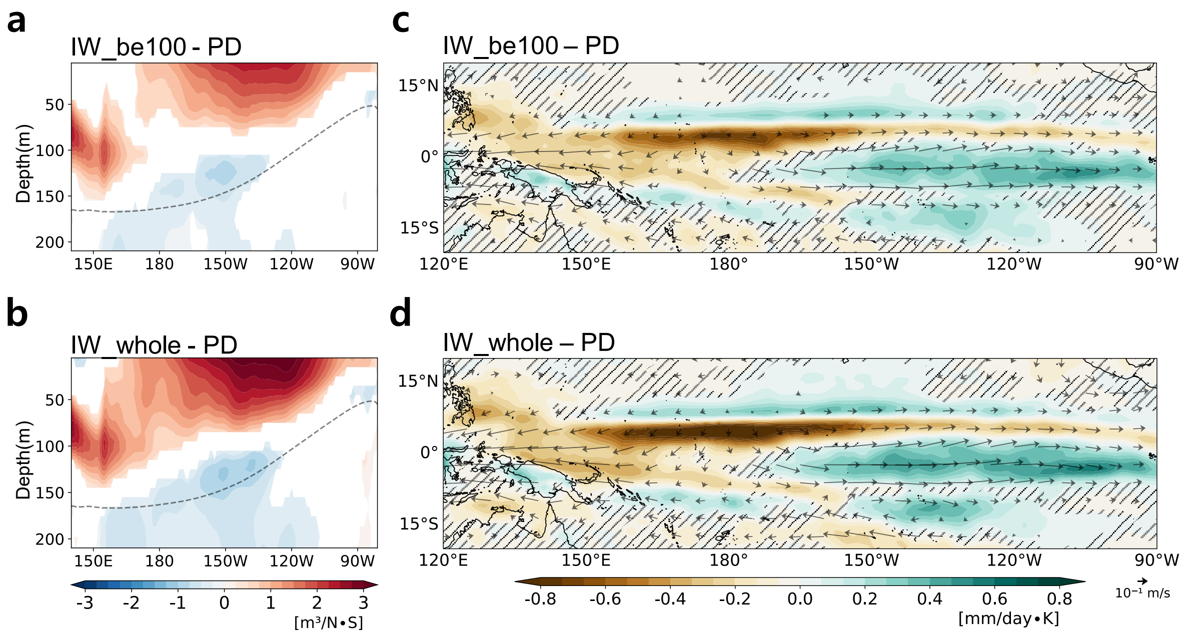


**Supplementary Fig. 12 | The changes in ENSO feedback.** The difference of zonal current regressed onto averaged wind stress over the equatorial Pacific (120°E–90°W, 5°S–5°N) during DJF from the PD period to **a** IW_be100 and **b** IW_whole. Only significant values at the 95% confidence level using the bootstrap test are shown. The difference of precipitation (shading) and 850hPa wind (vector) regressed onto Niño3 SSTA during DJF from the PD period to **c** IW_be100 and **d** IW_whole. Stippled areas are regions that are not significant at the 95% confidence level using the bootstrap test.

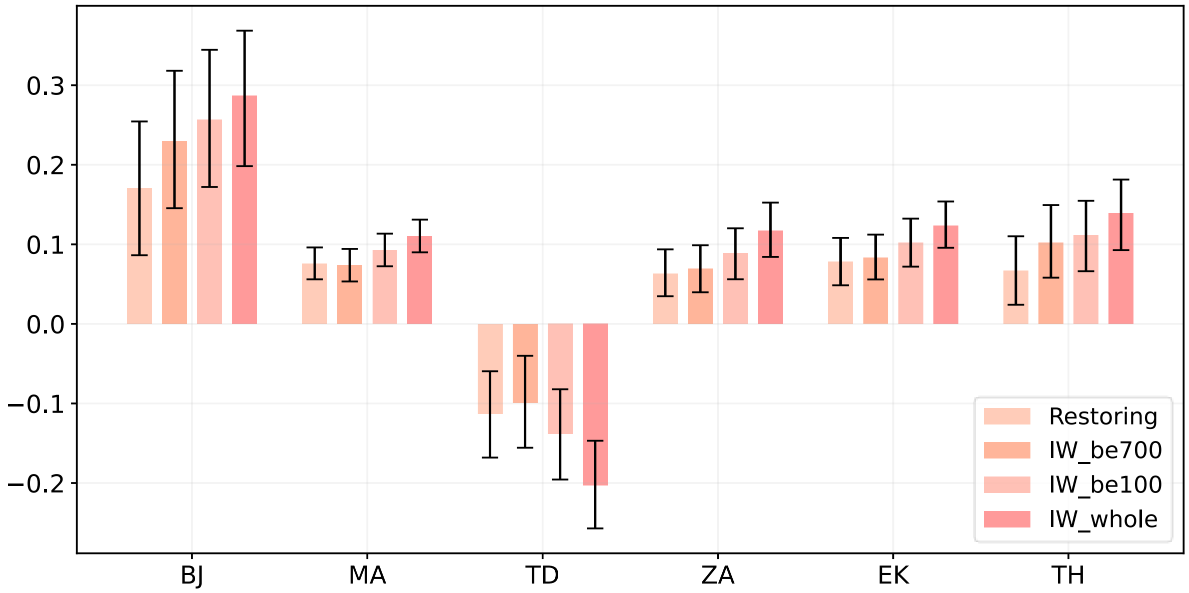


**Supplementary Fig. 13 | The changes in Bjerknes stability (BJ) index.** The difference BJ index calculated for the equatorial Pacific (150°E–90°W, 5°S–5°N) and its each term from the PD period to restoring, IW_be700, IW_be100 and IW_whole, respectively. From left to right, BJ index, mean advection (MA), thermal damping (TD), zonal advection feedback (ZA), Ekman feedback (EK), and thermocline feedback (TH) are shown. Error bars represent the 95% confidence interval using the bootstrap test.

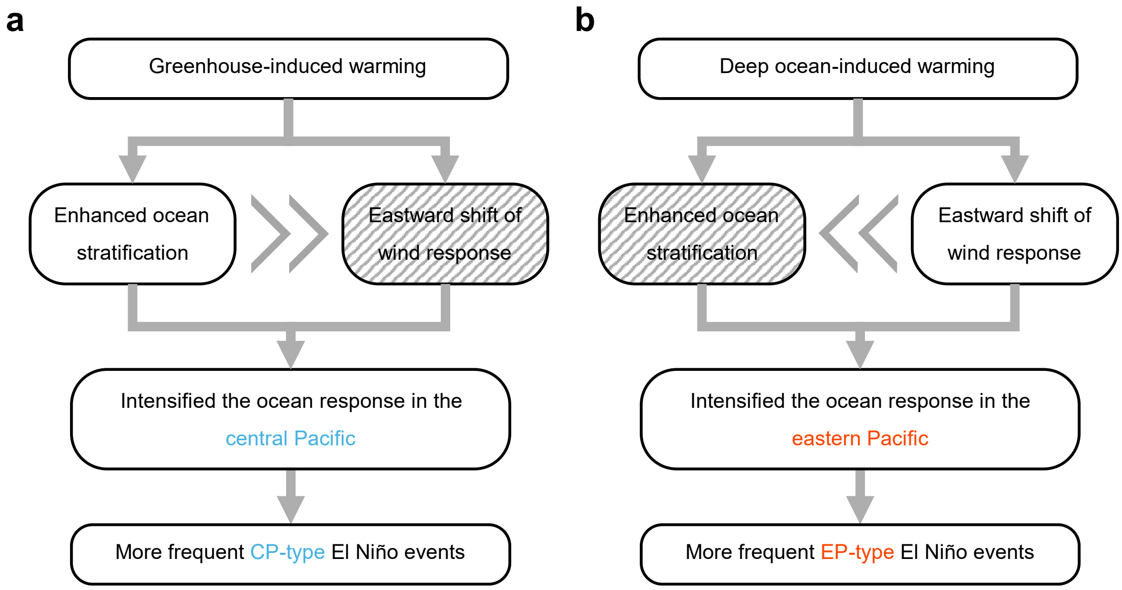


**Supplementary Fig. 14 | The schematic chart for the mechanism of the changes in ENSO.** Mechanisms for El Niño changes induced by **a** the greenhouse and **b** deep ocean warming. The dominant process is indicated by a greater-than symbol, while the other is stippled. Greenhouse gas warming increases ocean surface temperatures, leading to increased ocean stratification, and consequently intensifies the ocean response in the central Pacific, promoting more frequent CP-type El Niño events. Deep ocean warming begins from a state of initially reduced stratification, limiting its stratification effect. El Niño-like warming response to deep ocean warming contributes to an eastward shift in the wind response, which reinforces the ocean response in the eastern Pacific, culminating in a higher frequency of EP-type El Niño events.

**Supplementary Table 1 | The definition of parameters in the BJ index and the associated response sensitivity coefficients.** $\langle A \rangle_E$ and $[A]$ indicate quantities that are area-averaged over the eastern boxed region and over the equatorial Pacific basin, respectively.

| | Parameter | Balance equations |
|---|---|---|
| $u, v, w$ | The zonal, meridional and vertical current | |
| $T, T_{sub}$ | The ocean termperautre averaged to 45m and at 55m | |
| $\tau_x$ | The zonal wind stress | |
| $h$ | The thermocline depth | |
| $L_x, L_y$ | Longitudinal and latitudinal length of the eastern box | |
| $a_1, a_2$ | Anomalous SSTs averaged at the boundaries and over the area of a region | $\langle Q \rangle_E = -\boldsymbol{\alpha_s} \langle T \rangle_E$ |
| $\alpha_s$ | Thermodynamic damping | $[\tau_x] = \boldsymbol{\mu_a^*} \langle T \rangle_E$ |
| $\mu_a$ | A wind response over the eastern Pacific to SST forcing | |
| $\mu_a^*$ | A wind response over the equatorial Pacific to SST forcing | $\langle \tau_x \rangle_E = \boldsymbol{\mu_a} \langle T \rangle_E$ |
| $\beta_u$ | A response of an ocean surface zonal current to wind forcing | $\langle u \rangle_E = \boldsymbol{\beta_u} [\tau_x]$ |
| $\beta_w$ | A response of an ocean upwelling to a wind forcing | $\langle H(\overline{w}) w \rangle_E = -\boldsymbol{\beta_w} [\tau_x]$ |
| $\beta_h$ | A response of a zonal slope of the thermocline to wind forcing | $\langle h \rangle_E - [h] = \boldsymbol{\beta_h} [\tau_x]$ |
| $a_h$ | A response of an ocean subsurface temperature to thermocline change | $\langle H(\overline{w}) T_{sub} \rangle_E = \boldsymbol{a_h} \langle h \rangle_E$ |
| $\partial \overline{T} / \partial x$ | The horizonal mean temperature gradient | |
| $\partial \overline{T} / \partial z$ | The vertical mean temperature gradient | |
| $H(x)$ | Step function $\{1\ x \geq 0; 0\ x < 0\}$ | |
| $Q$ | Net anomalous heat flux | |

**Supplementary Table 2 | CMIP6 8 models list.**

| Model number | Model name | Model year (pi-control) | Model year (restoring) |
|---|---|---|---|
| 1 | ACCESS-ESM1-5 | 1000 | 620 |
| 2 | CESM2 | 1200 | 60 |
| 3 | CNRM-ESM2-1 | 500 | 60 |
| 4 | CanESM5 | 1051 | 60 |
| 5 | GFDL-ESM4 | 500 | 60 |
| 6 | MIROC-ES2L | 500 | 362 |
| 7 | NorESM2-LM | 501 | 119 |
| 8 | UKESM1-0-LL | 1880 | 510 |

**Supplementary References**


1. Jin, F.-F., Kim, S. T. & Bejarano, L. A coupled-stability index for ENSO. *Geophysical Research Letters* **33**, (2006).
2. Kim, S. T. & Jin, F.-F. An ENSO stability analysis. Part I: results from a hybrid coupled model. *Clim Dyn* **36**, 1593–1607 (2011).
3. Kim, G.-I. & Kug, J.-S. Process-Based Analysis of El Niño–Southern Oscillation Decadal Modulation. *Journal of Climate* **35**, 4753–4769 (2022).
4. Keller, D. P. *et al.* The Carbon Dioxide Removal Model Intercomparison Project (CDRMIP): rationale and experimental protocol for CMIP6. *Geoscientific Model Development* **11**, 1133–1160 (2018).
